# Perspectives of the Application of Liquid Biopsy in Colorectal Cancer

**DOI:** 10.1155/2020/6843180

**Published:** 2020-03-09

**Authors:** Yuhan Ding, Wenxia Li, Kun Wang, Chang Xu, Mengdi Hao, Lei Ding

**Affiliations:** ^1^Department of Oncology, Beijing Shijitan Hospital, Capital Medical University, Beijing 100038, China; ^2^Ninth School of Clinical Medicine, Peking University, Beijing 100038, China

## Abstract

Colorectal cancer (CRC) is one of the most common gastrointestinal tumors and the second leading cause of cancer death worldwide. Since traditional biopsies are invasive and do not reflect tumor heterogeneity or monitor the dynamic progression of tumors, there is an urgent need for new noninvasive methods that can supplement and improve the current management strategies of CRC. Blood-based liquid biopsies are a promising noninvasive biomarker that can detect disease early, assist in staging, monitor treatment responses, and predict relapse and metastasis. Over time, an increasing number of experiments have indicated the clinical utility of liquid biopsies in CRC. In this review, we mainly focus on the development of circulating tumor cells and circulating tumor DNA as key components of liquid biopsies in CRC and introduce the potential of exosomal microRNAs as emerging liquid biopsy markers in clinical application for CRC.

## 1. Introduction

Colorectal cancer (CRC) is one of the most common gastrointestinal tumors and the second leading cause of cancer death worldwide [1]. It is estimated that more than 1.8 million new CRC cases and 881,000 deaths occurred worldwide in 2018 [1]. In China, the incidence and mortality of CRC have increased in the past decade, as a result of the insidious nature of CRC, late diagnosis, and limited treatment options. Currently, the management of CRC relies primarily on serum biomarker levels, tissue biopsy, and imaging findings. However, the diagnostic accuracy and sensitivity of pathological and imaging methods are still limited, while the specificity and diagnostic performance of common serum markers are poor. Therefore, finding a powerful method to manage CRC in the long-term is crucial.

**Figure 1:**
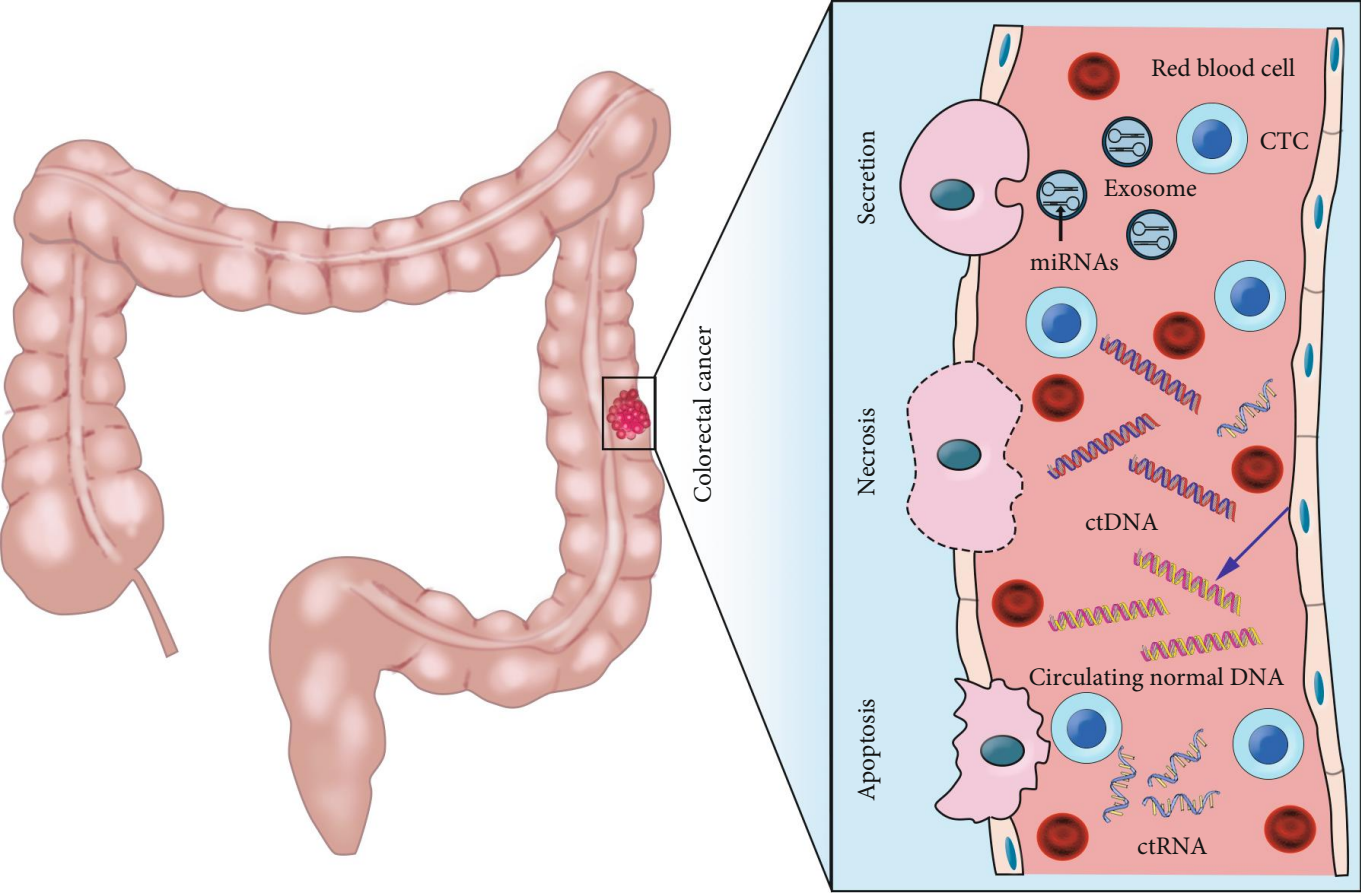
Circulating tumor cells (CTCs), circulating tumor DNA (ctDNA), circulating tumor RNA (ctRNA), and exosomes are promising liquid biopsy markers for colorectal cancer. CTCs from colorectal cancer can be shed from the primary tumor into the bloodstream, which also contains ctDNA released from tumor tissue through apoptosis, necrosis, and secretion, as well as circulating normal DNA released from healthy tissue. MicroRNAs (miRNAs) encapsulated by exosomes can be actively secreted into the extracellular fluid by various types of cells in the tumor or passively released due to the apoptosis and necrosis of tumor cells and can eventually be found in the circulation.

**Figure 2:**
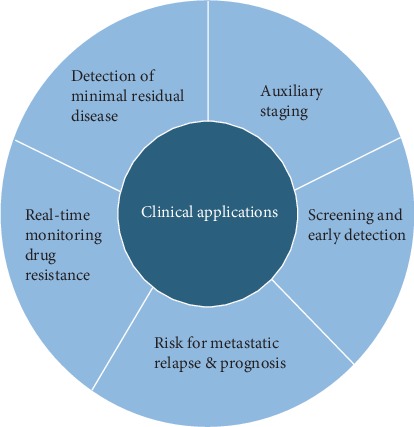
The multifaceted qualities of liquid biopsies demonstrate its potential clinical applications in the management of colorectal cancer.

In the past few years, a new diagnostic concept, liquid biopsy, has received widespread attention [2–4]. Liquid biopsy is a general term originally introduced in the analysis of circulating tumor cells (CTCs) [5] that now widely refers to the analysis of various biological fluids isolated from cancer patients, such as peripheral blood, urine, pleural effusion, ascites, and cerebrospinal fluid [6, 7]. However, peripheral blood remains the main source of fluid biopsy, and its analytes mainly include CTCs, circulating tumor DNA (ctDNA), circulating tumor RNA (ctRNA), and exosomes (Figure 1) [8]. Analysis of these blood components can be used for early cancer detection, auxiliary staging, prognosis assessment, and monitoring drug resistance and minimal residual disease (MRD) [9], highlighting the potential of liquid biopsies (Figure 2).

The molecular pathogenesis of CRC is extremely complex and heterogeneous. At present, the pathological features of CRC depend on biopsy or surgical specimens. However, due to its invasive nature, biopsies cannot always be performed routinely. The information obtained from a single biopsy provides only a limited snapshot of the tumor and fails to reflect heterogeneity. To some extent, liquid biopsies can compensate for the lack of traditional detection, track the evolutionary dynamics and heterogeneity of the tumor in real time, and provide a genetic overview of tumor lesions and dynamic information on genome evolution [10]. In addition, the analysis of therapeutic targets and drug-resistant gene mutations released into the circulation by CTCs and ctDNA could help to better elucidate and clinically manage drug resistance in cancer patients. To date, CTCs and ctDNA, as important components of liquid biopsies, have made good progress in the diagnosis, prognosis, and treatment of CRC. Exosomal microRNAs (miRNAs) are also considered to have great potential in the management of CRC as emerging biomarkers for liquid biopsies. In this review, we will outline the current state of liquid biopsy and its role in CRC management.

## 2. Circulating Tumor Cells

### 2.1. Origin

CTCs are tumor cells from the primary tumor or metastases that enter into the blood circulation [11, 12]. A number of CTCs could escape the body's immune recognition and drug treatment, find a suitable microenvironment in the body, and form a “seed” to grow in the distal tissue or primary tissue, causing tumor metastasis or recurrence [13].

The discovery of CTCs in the blood can be traced back to 1869 by Thomas Ashworth, an Austrian physicist, who found that patients with metastatic tumors may have some cells in the bloodstream that are homologous to the original tumor tissue. Limited by the testing methods available at the time, the discovery did not attract much attention [14]. In 1955, another report demonstrated the presence of CTCs in circulating blood [15]. Although their discovery was more than a century ago, CTCs have not entered clinical practice, mainly because the challenge of isolating these extremely rare cells from peripheral blood prevents the understanding of their clinical significance.

### 2.2. Enrichment of CTCs

The number of CTCs detected in the peripheral blood is extremely small, with approximately 1 CTC in 10^7^ white blood cells, so the accurate detection of CTCs is crucial [12]. In principle, there are various methods for detecting CTCs on the basis of biological or physical properties [16, 17].

Biotechnology mainly refers to immunomagnetic separation. Immunomagnetic separation technology combines cell surface-based antigens with magnetic beads attached to specific antibodies and enriches cells under the action of an external magnetic field [18]. The cell search system uses magnetic beads to bind specific antigens to separate epithelial cells in the blood, and its main principle is to separate CTCs by screening CK+, DAPI+, and CD45- cells [19]. The system is the only CTC detector that has been approved by the US Food and Drug Administration (FDA) for clinical studies in patients with breast, colorectal, and prostate cancer [20–23]. In the study of metastatic CRC (mCRC) using the cell search system, the progression-free survival (PFS) and overall survival (OS) times of patients with ≥3 CTCs/7.5 mL blood were shorter than those of patients with <3 CTCs (*P* < 0.0001), suggesting that the number of CTCs was an independent predictor of PFS and OS in patients with mCRC [24]. Nevertheless, due to the possibility of epithelial-mesenchymal transition in tumor cells and the lack of widely and commonly expressed markers on the cell surface of nonepithelial solid tumor cells, this method still has technical bottlenecks, coupled with its high price, which make it not routinely used in CTC detection [25].

CTCs are separated by density, size, and deformability according to the physical characteristics of CTC enrichment technology [25]. CTCs separated by density gradient centrifugation, membrane filtration separation, microfluid detection, and other physical methods will not damage the structure of the cells, and the separated cells can continue to be used in immunohistochemistry or immunofluorescence assays and other related studies [26–28]. However, these methods have poor specificity, low sensitivity, and poor stability, making obtaining tumor cells with small sizes and spontaneous and pressurized changes in shape difficult, leading to a false-positive rate due to the capture of blood cells [16].

To solve the shortcomings of the above methods, several new detection methods have been developed in recent years. In April 2017, at the annual meeting of the American Association for Cancer Research (AACR), the method of diagnostic leukapheresis was introduced to separate CTCs [29], which is not only of high quantity but also of high quality and can be used for subsequent diagnostic analyses [30]. Recently, the Hydro-Seq technology developed by Cheng et al. [31] can accurately separate CTCs from patients' blood samples with ultrahigh purity without the contamination of white blood cells and red blood cells, and the comprehensive analysis of CTCs can be conducted with high throughput and without contamination, which can effectively provide treatment plans for patients in clinical practice.

## 3. Circulating Tumor DNA

### 3.1. Origin

ctDNA is a kind of double-stranded DNA fragment derived from tumor cells, ranging in size from 0.18 to 21 kb. It is mainly found in bloodstream, synovial fluid, cerebrospinal fluid, and saliva and can be excreted through urine and feces, with extremely small content [32–34]. The presence of ctDNA could date back to early studies of circulating free DNA (cfDNA). In 1948, Mandel and Metais first reported the presence of circulating nucleic acids in cells in human blood [35]. cfDNA is considered to originate mainly from necrotic or apoptotic cells [36]. Thirty years later, Leon et al. found that the level of cfDNA in the serum of individuals with cancer was higher than that of the healthy control group, and it had the genomic characteristics of tumor cells [37]. Cell turnover generally increased as tumor size increased. As a result, cancer patients have much higher levels of cfDNA than healthy people. In other physiological conditions or clinical cases, the concentration of cfDNA also increases, such as exercise [38], infection [39], cerebral infarction [40], acute trauma [41], and transplantation [42]. Afterwards, in 1989, it became clear that cfDNA is partly derived from tumors [43]. ctDNA is a fraction of cfDNA that is released from tumor cells into the blood, and in principle, it carries the same specific mutations as the corresponding cancer cells, such as tumor proto-oncogenes and oncogene mutations, microsatellite alterations, and DNA methylation [44].

### 3.2. Detection of ctDNA

The extraction efficiency of ctDNA is not satisfactory due to its small fragment size, low content, and easy combination with plasma protein. To overcome these limitations, developing sensitive and repeatable methods to identify ctDNA is crucial.

The common ctDNA detection technology is based on two major platforms, one of which is PCR. The qPCR-based methods are widely used to detect gene mutations in cfDNA; however, the sensitivity is not up to 0.1%. Recently, improved qPCR-based methods have become feasible. For example, allele-specific qPCR has been devised to detect hotspot mutations in plasma and serum of cancer patients with a sensitivity between 0.014% and 0.004% [45]. In parallel, dPCR was found to have higher sensitivity to identify genomic changes. In 1999, Vogelstein et al. [46] introduced the dPCR method, which makes it possible to accurately identify and quantify rare mutant fragments and is widely used to quantitatively determine ctDNA levels [47–50]. For example, picodroplet-based dPCR has been reported to accurately detect one mutant *KRAS* gene in more than 200,000 wild-type *KRAS* genes [51]. However, the ability of dPCR to perform dynamic analysis of a single mutation is based on prior knowledge of the mutant allele [52].

Next-generation sequencing- (NGS-) based technologies are a massively parallel sequencing technique that can analyze larger targets. It can not only detect known genes but also analyze large parts of the genome unknowingly and identify multiple mutations with greater sensitivity [53, 54]. Due to the fast speed and high throughput of NGS and the small amount of DNA samples required, it has been increasingly applied in the clinical field of cancer, such as gene panel sequencing, whole-exome sequencing, and whole-genome sequencing [55–59]. Murtaza et al. showed that cfDNA sequencing from serial plasma samples can be used to characterize the evolutionary genomic map of entire exon mutations [60]. Whole-genome sequencing of cfDNA released from tumor cells into blood has been demonstrated to recognize tumor mutations, such as focal amplification [61], gene rearrangement [62], and chromosomal aberrations [63]. Advances in NGS technology have expanded the ability to detect cancer mutations in blood and enriched the clinical application of ctDNA-based liquid biopsies. Compared to dPCR, the ability to analyze larger target regions is also at the expense of lower sensitivities to detect rare mutations within 0.1% of the mutated DNA fragment or slightly less than 0.1% [64].

In light of the advantages and disadvantages of both dPCR and NGS, they can complement each other in practical clinical applications.

## 4. Clinical Application of CTCs and ctDNA in CRC

As promising biomarkers, CTCs and ctDNA have great appeal to researchers who attempt to detect tumor-specific changes in cancer progression in real time and show their potential for early detection, auxiliary staging, prognostic assessment, and monitoring of the drug resistance and MRD in CRC (see Tables 1 and 2).

### 4.1. CTCs and ctDNA for CRC Screening

The onset of CRC is insidious, and more than 80% of patients are already in the middle and late stages when they are diagnosed, even with liver and lung metastasis. Moreover, the 5-year survival rate of patients with advanced CRC is still low, so the early detection of tumors based on blood markers provides benefits for improving the survival rate of CRC patients.

There are few studies on CTC detection for the screening of CRC. In a recent prospective study, CTCs were tested and counted in 620 patients (including healthy individuals, stage I-IV CRC patients, and precancerous lesions), and the data showed that the overall accuracy of CTC detection for all colorectal disease stages, including precancerous lesions, was 88% [65]. In addition, the isolation and count of circulating endothelial cell clusters derived from tumors in CRC may provide a new perspective for distinguishing patients with early-stage colon cancer from healthy individuals [66].

One of the early events of carcinogenesis is epigenetic changes, including DNA methylation and histone modifications. ctDNA also reflects the epigenetic characteristics of cancer patients and helps in the early diagnosis of cancer by detecting epigenetic changes [67]. Clinical data indicate that circulating *SEPT9* DNA as a methylation marker is as sensitive as 87% in detecting stage I CRC, 84% in detecting stage II CRC, and 90% in overall CRC [68]. Two recent studies have shown that tumor-specific methylation changes can be detected in plasma two years before tumor diagnosis [69, 70]. Further research by Guo et al. showed that ctDNA methylation changes in plasma could not only be applied in the screening of tumors but also reveal the tissue source of tumors [71].

As outlined above, the detection of CTCs and ctDNA is challenging in early colon cancer screening, but more prospective experiments will validate the utility of these blood-based noninvasive procedures in a patient's physical examination.

### 4.2. CTCs and ctDNA Detection for the Auxiliary Staging of CRC

Tumor cells in the circulation have the ability to proliferate or migrate, thereby providing a reliable means for neoplasm staging [72]. Several studies have shown that counting CTCs reflects the patient's tumor burden to some extent and that in advanced CRC, the detection rate of CTCs increased with the increase in tumor stage [73]. Further investigations on the tumor TNM staging system revealed that the number of CTCs detectable was positively correlated with primary tumor size and depth of invasion, lymph node invasion, and distant metastasis, suggesting that CTCs are feasible for judging lymph node infiltration and distant metastasis [74–76].

Quantifying ctDNA levels is closely related to cancer stage and tumor burden [77]. In a recent study that analyzed ctDNA in patients with CRC at different stages, Dr. Yang et al. clarified that the ctDNA concentration in stage I patients was significantly lower than that in stage IV patients and that the ctDNA concentration was positively correlated with tumor size [78]. The observations and evaluations made during a study on the ctDNA analysis of patients receiving cetuximab suggested that the *KRAS* mutations in plasma detected by ctDNA were not detected by the radiological method until 10 months later [79]. These results support the idea that ctDNA detection can complement the traditional cancer screening methods and provide a basis for cancer staging, depending on the specificity and sensitivity of its diagnosis.

CTCs and ctDNA detection, as the link between tumor metastasis and the primary tumor, can reflect cancer progression in real time and have a reference value for neoplasm staging. Some scholars have suggested integrating the blood-based liquid biopsy into the existing TNM staging system and proposed the new concept of “TNMB,” where “B” refers to blood, to enhance the existing TNM staging system for the diagnosis and classification of conventional cancers [80].

### 4.3. CTCs and ctDNA as Prognostic Markers in CRC

Current research has confirmed that CTCs detected in blood can be used as an independent prognostic factor for tumors such as prostate cancer [81], breast cancer [82], and colon cancer [83, 84]. The higher the number of CTCs detected, the worse the prognosis of patients. PFS and the median OS are significantly shortened in patients with ≥3 CTCs/7.5 mL of mCRC [20]. In a study of 183 patients with CRC, blood samples were collected at various time points before and during the follow-up. From the data collected during the study, the authors concluded that preoperative CTC was associated with a significant reduction in patient survival and was able to identify patients at high risk of recurrence [85]. Many studies have also shown the value of the positive detection CTCs for poor prognosis in CRC patients [86, 87]. Interestingly, a prospective study in 2015 came to a completely different conclusion from the findings above [88]. In this study, peripheral blood samples from 519 patients with stage III CRC after tumor resection were examined for CTCs, and no clear correlation was found between the presence of CTCs and the survival of CRC patients. This controversial result is probably due to the different metastatic modes of CRC and the low level of CTCs detected, which may require longer follow-up for verification. In addition to increasing the amount of blood samples to increase the amount of CTCs detected, the study can also be improved by replacing peripheral blood with mesenteric venous blood. Deneve and his colleagues demonstrated that more CTCs were detected in mesenteric blood than peripheral blood, and the follow-up analysis showed that patients with high CTC counts had a poor prognosis [89].

The level of ctDNA can indicate its prognostic value [90]. Recent studies have found that monitoring ctDNA levels in CRC patients can show disease recurrence and response to treatment earlier than traditional tumor markers or radiologic diagnosis [91, 92]. In a retrospective study of 97 mCRC patients, higher levels of cfDNA mutations detected were linked with significantly worse OS and higher mutation loads [93]. Dr. El et al. reported that a high cfDNA level could be an independent prognostic factor for shorter OS [93]. Another study of 96 patients with stage III colon cancer showed that ctDNA was detected in postoperative blood samples from 20 patients, while ctDNA remained after adjuvant chemotherapy in 17 patients. In all 96 patients with stage III colon cancer, patients with detectable ctDNA levels differed significantly from those without ctDNA in terms of recurrence-free survival [94]. A systematic review including 10 studies found that high ctDNA levels before treatment are related to shorter survival in mCRC patients [95]. Moreover, *KRAS* mutations detected in fresh plasma have been reported as an indicator of poor prognosis in CRC patients [96].

### 4.4. CTCs and ctDNA for Monitoring Drug Resistance and Guiding Medication

The *RAS* gene needs to be detected in patients with CRC to determine the follow-up treatment plan. Compared with gene detection in tissue, ctDNA has a very obvious advantage in guiding targeted drugs. ctDNA detection is a noninvasive method that can overcome tumor heterogeneity, and it has high sensitivity and specificity. Bettegowda et al. [97] used peripheral blood ctDNA to detect mutations of the *KRAS* gene in 206 colon cancer patients and found that the sensitivity reached 87.2% and the specificity was as high as 99.2%. Other studies have shown that the later the tumor stage is, the higher the detection rate of ctDNA, and the sensitivity of patients in stage IV was close to 100% [98].

Currently, anti-EGFR therapy is now approved for wild-type *RAS* colorectal tumors [99]. In addition, *BRAF* mutations are generally considered to be another biomarker for the resistance to cetuximab and panitumumab single antigen [100]. Together, *KRAS* and *BRAF* are considered effective predictors of anti-EGFR therapy. In past studies, there was an inconsistency in the detected gene mutations between blood and tissues. When the tissue was detected as wild-type *KRAS*, the peripheral blood was detected as mutant-type *KRAS*. ctDNA analysis in vivo and in vitro assays have shown that by blocking the EGFR pathway, *KRAS* and *NRAS* mutations will occur rapidly, and mutations can usually be detected before imaging confirms tumor recurrence [101]. ctDNA has been observed to change in patients receiving panitumumab or cetuximab for the treatment of mCRC; blocking the EGFR pathway leads to the production of *KRAS* mutant clones, and the clones gradually decreased after stopping the drug. After a period of time, tumor cells were able to restore drug sensitivity again, suggesting that clonal evolution persisted, and ctDNA can be used to dynamically monitor *KRAS* mutation levels, providing a basis for reapplication of anti-EGFR drugs [102].

In addition, CTCs isolated and enriched in CRC patients have been reported to also detect the presence of *KRAS* and *BRAF* hotspots [103]. In a study of 44 early-stage and late-stage CRC patients and 18 healthy individuals, CTCs were isolated from the blood through microsieve filtration to screen *KRAS* and *BRAF* mutations in CRC patients. The results suggested that tumor tissue and CTCs had 70% identity in the *KRAS* mutation state, while the *BRAF* mutation was less consistent [104]. Kalikaki et al. evaluated *KRAS* mutations in continuous plasma samples from 31 mCRC patients and found that the CTCs of individual patients exhibited different *KRAS* mutation states during treatment [105]. Encouragingly, a recent innovative experiment showed the potential of CTCs to predict drug resistance [106]. The study first detected gene copy number aberrations in 88 CTC cells isolated from 13 small cell lung cancer patients and generated a classifier based on the copy number aberrations in CTCs. The classifier was then tested on 6 patient-derived CTC explant tumors and 112 CTC samples from 18 additional patients, and the classifier accuracy was found to be 83.3%. Moreover, significant differences were observed in PFS among patients classified by chemotherapy resistance. Thus, a molecular diagnostic method based on CTCs has been developed to determine whether a patient is sensitive or tolerant to chemotherapy.

In summary, ctDNA and CTCs, with their characteristics of easy access and overcoming the spatial heterogeneity of tumors, can be used to vertically detect the mutation status of tumor patients and tailor individualized treatment according to the molecular characteristics of patients' tumors, thus showing a promising application prospect in guiding the targeted treatment of CRC.

### 4.5. CTCs and ctDNA as Monitoring Tools for MRD

At present, conventional surveillance modalities of patients with stage II and III CRC after therapeutic surgery attempt to detect MRD in real time. However, the sensitivity of serum carcinoembryonic antigen tests and tomographic scans to the detection of micrometastatic disease is not high, and computed tomography (CT) scans carry the risk of frequent exposure to contrast agents and radiation, so identifying MRD in real time is difficult. Some studies have shown that ctDNA has a short half-life of approximately two hours, which can reflect the tumor status in real time and potentially detect the presence of MRD prior to radiological diagnosis [107, 108].

In a prospective study involving 230 patients with stage II colon cancer who underwent therapeutic surgery, the role of ctDNA in detecting MRD was confirmed [109]. Among patients not treated with chemotherapy, radiological recurrence was detected during follow-up in only 9.8% of patients who were ctDNA-negative postoperatively, while 78% of ctDNA-positive patients relapsed after surgery. The presence of ctDNA in other chemotherapy-treated patients was also associated with poor recurrence-free survival. More recently, serial plasma samples from patients with locally advanced rectal cancer during multimodality treatment have been used to provide evidence that the presence of ctDNA after chemoradiotherapy or after surgery shows a significant reduction in recurrence-free survival [110]. A number of researchers have also reported the role of ctDNA in predicting recurrence, and CRC patients with positive postoperative ctDNA have a higher risk of MRD, while negative postoperative ctDNA may provide assurance of disease control [111–113].

CTC levels appear to be connected with poor postoperative survival and disease recurrence in CRC patients [114–116]. In a recent prospective study of 44 patients with CRC with liver metastasis, patients with CTC-positive detected preoperatively had all recurred during the postoperative follow-up, and recurrence occurred in 65% of patients who were CTC-negative [117]. Moreover, CTC assays are expected to supplement imaging methods for the diagnosis of disease recurrence. Another significant study of 84 colon cancer patients undergoing chemotherapy showed that changes in the number of CTCs reflected the objective efficacy of the tumor at an early stage, with a sensitivity of 64%, specificity of 70%, and positive predictive value of 74%. Therefore, CTCs have the potential to detect MRD earlier than imaging responses [118].

## 5. Emerging Liquid Biopsy Markers

Although the application of liquid biopsy in oncology has emerged and developed at an incredible speed, the information extracted from ctDNA and CTCs still cannot fully meet the requirements of tumor management, so expanding the range of analytes examined is expected to help liquid biopsy reach its potential in clinical application. Biomarkers such as ctRNA and platelets while patients are undergoing tumor therapy are also candidates for fluid biopsy [119–125], while exosomal miRNAs may have the potential to make more contributions to the development of this field in the near future.

Exosomes are extracellular vesicles with a diameter of 30-100 nm secreted from various cells under normal physiological and pathological conditions [126]. Exosomes contain a variety of molecules, including proteins, lipids, and nucleic acids (such as DNA, mRNAs, miRNAs, and lncRNAs), the contents of which reflect the physiological or pathological conditions of the host cells [127]. The biological function of cancer-derived exosomal miRNAs in the genetic transfer between cancer cells has been gradually confirmed and has become a hot spot in cancer research [128, 129]. An increasing number of exosomal miRNAs have been found to play an important role in the diagnosis and treatment of CRC, as shown in Table 3.

Growing evidence suggests that exosomal miRNAs are potent mediators of cell communication, supporting the progression and metastasis of CRC [130–133]. Circulating exosomal miR-25-3p in CRC has been shown to be involved in cancer metastasis, inducing vascular permeability and angiogenesis by targeting KLF2 and KLF4 to regulate the expression of VEGFR2, ZO-1, occludin, and Claudin-5 in endothelial cells. Furthermore, the expression level of miR-25-3p in circulating exosomes was significantly lower in CRC patients without metastasis than in CRC patients with metastasis [131]. Similarly, Zhang et al. demonstrated that exosomal miR-200b can amplify proliferation factors to adjacent or distant cells to promote the proliferation of CRC cells and achieve effective tumor growth [132].

More recently, exosomal miRNA content has emerged as a potential biomarker of CRC. A study on the analysis of serum exosomes from 133 CRC patients and 60 healthy individuals found that the decreased expression of serum exosomal miR-150-5p was closely related to poor differentiation, positive lymph node metastasis, and TNM progression. Serum extracellular miR-150-5p has been confirmed as an independent prognostic indicator of CRC, and the survival time of patients with a low expression of serum extracellular miR-150-5p was significantly longer than that of patients with high expression [134]. Similar results were obtained in serum, where high levels of exosomal miR-6803-5p have been shown to be associated with the poor prognosis of CRC [135]. Another study revealed that, compared with healthy individuals in the control group, the expression levels of miR-19a and miR-92a in the serum of patients with CRC are significantly increased, which is associated with CRC recurrence [136].

Additionally, the potential of miRNAs to treat CRC resistance is noteworthy. As described by Jin et al. in 2019, the expression levels of miR-21-5p, miR-1246, miR-1229-5p, and miR-96-5p in the serum exosomes of the chemosensitive control group were lower than those of chemosensitive patients, suggesting that the above miRNAs of exosomes can predict the chemical resistance of CRC patients and are expected to be new targets for the treatment of drug resistance [137].

Overall, exosomal miRNAs are a complementary tool for fluid biopsy in CRC applications and are very attractive. Nevertheless, their use is still hampered by a number of technical problems that need to be overcome [138]. The low purity of exosomal miRNAs may be caused by irregular sample collection, pretreatment, storage and transportation, or differences in vesicle counting methods. Another key factor that needs to be taken into consideration is the level of platelet products in clinical blood samples [139]. Moreover, the tumor specificity of miRNAs is also a challenge in clinical use. For instance, some studies [140–143] have shown that miR-10b is involved in the development of various tumors, including breast cancer, pancreatic cancer, and CRC, which reveals that endogenous normalizers may be needed to quantify the expression of exosomal miRNAs in CRC. Therefore, further studies are needed to reveal the exact role of exosomal miRNAs in the clinical application of CRC.

## 6. Conclusion

In summary, liquid biopsy is a growing noninvasive method. The term liquid biopsy is used to refer more to CTCs but is now also associated with ctDNA and other biomarkers such as miRNAs. With the rapid development of liquid biopsy in oncology research, this method can be used for CRC screening and early detection and provide more evidence for the clinical staging of patients diagnosed with CRC. In addition, it provides prognostic and predictive data that can be used to monitor MRD and combat drug resistance. Although a large number of clinical studies of liquid biopsy for CRC have been carried out and promising preliminary results have been obtained, the road to the clinic is not free from hurdles.

First, the biological basis of ctDNA remains controversial; apoptosis and necrosis are the most frequently discussed origins of ctDNA. The release mechanism of ctDNA is not fully understood at present, and studies suggest that autophagy and intermittent hypoxia may be closely related to the release of ctDNA [36]. In addition, false-positive results may occur during the detection phase of liquid biopsy due to the collection of benign circulating epithelial cells or blood cells. Therefore, there is an urgent need to establish standardized methods for sample collection, processing, and storage to eliminate differences between studies.

Liquid biopsy can dynamically monitor the progression of CRC and provide important information about tumor heterogeneity compared to the currently used biomarkers. Despite these advantages, the transfer of liquid biopsies from the laboratory to the clinical environment requires more multicenter, larger-scale, and longer-term studies to demonstrate its superiority. The clinical usefulness of liquid biopsies in CRC is expected to reach an accurate and clear consensus in the near future.

## Figures and Tables

**Table 1:** Application value of CTCs in CRC.

Reference	Biomarkers	Methods	Potential clinical utility
[66]	CD45	scrm PCR	Screening and early detection
[76, 85, 87]	EpCAM	CellSearch™	Prognostic
[83]	CD45	Cyttel	Predictive and prognostic
[89]	CK-19, EpCAM	CK19-Epispot and CellSearch™	Prognostic
[103]	EpCAM, *APC*, *KRAS*, *PIK3CA*	NGS, CGH, CellSearch™	Therapy and relapse monitoring
[105]	*KRAS*	PNA-based qPCR	Therapy monitoring
[114]	LGR5, EMT	CanPatrol™, mRNA ISH	Prognostic
[115]	CK20	CMx platform	Monitoring early recurrence
[116, 117]	EpCAM	CellSearch™	Predictive and prognostic

Abbreviations: scrm PCR: single-cell RNA and mutational analysis PCR; NGS: next-generation sequencing; CGH: array-comparative genomic hybridization; PNA-based qPCR: peptide nucleic acid-based real-time PCR; mRNA ISH: mRNA in situ hybridization.

**Table 2:** Application value of ctDNA in CRC.

Reference	Biomarkers	Methods	Potential clinical utility
[67, 68]	Methylated *SEPT9*	qPCR	Screening
[78]	*TP53*, *PIK3CA*, *APC*, *EGFR*	Targeted sequencing	Early diagnosis and auxiliary staging of CRC
[79]	*KRAS*	454 pyrosequencing, BEAMing	Therapy selection and monitoring
[92]	SSVs	ddPCR	Prognostic and monitoring
[94]	A panel of 15 genes	Safe-Seqs	Prognostic
[96]	*KRAS*, *PIK3CA*, *BRAF*	BEAMing	Prognostic
[97]	*KRAS*	dPCR	Prognostic and monitoring drug resistance
[98]	Mutation patterns and VAFs	NGS	Tumor burden monitoring
[99]	*KRAS*, *NRAS*	Bidirectional Sanger sequencing	Therapy selection and monitoring
[101]	*KRAS*	ddPCR	Monitoring drug resistance
[102]	*KRAS*	BEAMing, ddPCR, NGS	Therapy selection and monitoring
[107]	SSVs, SPMs	ddPCR	Detecting MRD and predicting recurrence
[109]	*TP53*, *APC*, *KRAS*	Safe-Seqs	Detecting MRD and predicting recurrence
[113]	Methylated *BCAT1* and *IKZF1*	Triplex real-time qPCR	Detecting MRD and therapy selection

Abbreviations: qPCR: real-time PCR; SSVs: somatic structural variants; ddPCR: droplet digital PCR; Safe-Seqs: Safe-Sequencing system; dPCR: digital PCR; NGS: next-generation sequencing; VAFs: variant allele frequencies; SPMs: somatic point mutations; MRD: minimal residual disease.

**Table 3:** Exosomal miRNAs as emerging liquid biopsy markers in CRC.

Exosomal miRNAs	Methods	Expression change	Potential clinical utility	Reference
miR-21	Amplification-free assay for electrochemical detection and qRT-PCR	Upregulation	Screening and prognostic	[130]
miR-25-3p	Microarray analysis and RT-PCR	Upregulation	Diagnostic and therapeutic	[131]
miR-200b	Northern blot and qRT-PCR	Upregulation	Diagnostic and therapeutic	[132]
miR-150-5p	qRT-PCR	Downregulation	Prognostic	[134]
miR-6803-5p	qRT-PCR	Upregulation	Diagnostic and prognostic	[135]
miR-19a, miR-92a	Microarray analysis and qRT-PCR	Upregulation	Prognostic	[136]
miR-21-5p, miR-1246, miR-1229-5p, miR-96-5p	qRT-PCR	Upregulation	Monitoring and treatment of chemoresistance	[137]

Abbreviations: qRT-PCR: quantitative real-time PCR; RT-PCR: reverse transcription PCR.

## References

[1] Bray F., Ferlay J., Soerjomataram I., Siegel R. L., Torre L. A., Jemal A. (2018). Global cancer statistics 2018: GLOBOCAN estimates of incidence and mortality worldwide for 36 cancers in 185 countries. *CA: a Cancer Journal for Clinicians*.

[2] Marcuello M., Vymetalkova V., Neves R. P. L. (2019). Circulating biomarkers for early detection and clinical management of colorectal cancer. *Molecular Aspects of Medicine*.

[3] Normanno N., Cervantes A., Ciardiello F., De Luca A., Pinto C. (2018). The liquid biopsy in the management of colorectal cancer patients: current applications and future scenarios. *Cancer Treatment Reviews*.

[4] Vymetalkova V., Cervena K., Bartu L., Vodicka P. (2018). Circulating cell-free DNA and colorectal cancer: a systematic review. *International Journal of Molecular Sciences*.

[5] Alix-Panabières C., Pantel K. (2013). Circulating tumor cells: liquid biopsy of cancer. *Clinical Chemistry*.

[6] Siravegna G., Marsoni S., Siena S., Bardelli A. (2017). Integrating liquid biopsies into the management of cancer. *Nature Reviews Clinical Oncology*.

[7] Wan J. C. M., Massie C., Garcia-Corbacho J. (2017). Liquid biopsies come of age: towards implementation of circulating tumour DNA. *Nature Reviews Cancer*.

[8] Heitzer E., Haque I. S., Roberts C., Speicher M. R. (2019). Current and future perspectives of liquid biopsies in genomics-driven oncology. *Nature Reviews Genetics*.

[9] Cohen J. D., Li L., Wang Y. (2018). Detection and localization of surgically resectable cancers with a multi-analyte blood test. *Science*.

[10] Gerlinger M., Rowan A. J., Horswell S. (2012). Intratumor heterogeneity and branched evolution revealed by multiregion sequencing. *The New England Journal of Medicine*.

[11] Masuda T., Hayashi N., Iguchi T., Ito S., Eguchi H., Mimori K. (2016). Clinical and biological significance of circulating tumor cells in cancer. *Molecular Oncology*.

[12] Hardingham J. E., Grover P., Winter M., Hewett P. J., Price T. J., Thierry B. (2015). Detection and clinical significance of circulating tumor cells in colorectal cancer—20 years of progress. *Molecular Medicine*.

[13] Lu Y., Lian S., Cheng Y. (2019). Circulation patterns and seed-soil compatibility factors cooperate to cause cancer organ-specific metastasis. *Experimental Cell Research*.

[14] Ashworth T. R. (1869). A case of cancer in which cells similar to those in the tumours were seen in the blood after death. *Australian Medical Journal*.

[15] Engell H. C. (1955). Cancer cells in the circulating blood; a clinical study on the occurrence of cancer cells in the peripheral blood and in venous blood draining the tumour area at operation. *Acta Chirurgica Scandinavica Supplementum*.

[16] Li Y., Wu S., Bai F. (2018). Molecular characterization of circulating tumor cells—from bench to bedside. *Seminars in Cell & Developmental Biology*.

[17] Ignatiadis M., Lee M., Jeffrey S. S. (2015). Circulating tumor cells and circulating tumor DNA: challenges and opportunities on the path to clinical utility. *Clinical Cancer Research*.

[18] Alix-Panabieres C., Pantel K. (2014). Challenges in circulating tumour cell research. *Nature Reviews Cancer*.

[19] Paterlini-Brechot P., Benali N. L. (2007). Circulating tumor cells (CTC) detection: clinical impact and future directions. *Cancer Letters*.

[20] Bidard F. C., Peeters D. J., Fehm T. (2014). Clinical validity of circulating tumour cells in patients with metastatic breast cancer: a pooled analysis of individual patient data. *The Lancet Oncology*.

[21] Riethdorf S., Fritsche H., Muller V. (2007). Detection of circulating tumor cells in peripheral blood of patients with metastatic breast cancer: a validation study of the CellSearch system. *Clinical Cancer Research*.

[22] Cristofanilli M., Hayes D. F., Budd G. T. (2005). Circulating tumor cells: a novel prognostic factor for newly diagnosed metastatic breast cancer. *Journal of Clinical Oncology*.

[23] Cristofanilli M., Budd G. T., Ellis M. J. (2004). Circulating tumor cells, disease progression, and survival in metastatic breast cancer. *The New England Journal of Medicine*.

[24] Cohen S. J., Punt C. J., Iannotti N. (2008). Relationship of circulating tumor cells to tumor response, progression-free survival, and overall survival in patients with metastatic colorectal cancer. *Journal of Clinical Oncology*.

[25] Arya S. K., Lim B., Rahman A. R. (2013). Enrichment, detection and clinical significance of circulating tumor cells. *Lab on a Chip*.

[26] Ankeny J. S., Court C. M., Hou S. (2016). Circulating tumour cells as a biomarker for diagnosis and staging in pancreatic cancer. *British Journal of Cancer*.

[27] Tien Y. W., Kuo H. C., Ho B. I. (2016). A high circulating tumor cell count in portal vein predicts liver metastasis from periampullary or pancreatic cancer: a high portal venous CTC count predicts liver metastases. *Medicine*.

[28] Poruk K. E., Valero V. R., Saunders T. (2016). Circulating tumor cell phenotype predicts recurrence and survival in pancreatic adenocarcinoma. *Annals of Surgery*.

[29] Fischer J. C., Niederacher D., Topp S. A. (2013). Diagnostic leukapheresis enables reliable detection of circulating tumor cells of nonmetastatic cancer patients. *Proceedings of the National Academy of Sciences of the United States of America*.

[30] Lambros M. B., Gil V. S., Crespo M. (2017). Abstract 993: diagnostic leukapheresis (DLA): molecular characterisation and organoid culture of circulating tumor cells (CTC) from metastatic castration resistant prostate cancer (mCRPC). *Cancer Research*.

[31] Cheng Y. H., Chen Y. C., Lin E. (2019). Hydro-Seq enables contamination-free high-throughput single-cell RNA- sequencing for circulating tumor cells. *Nature Communications*.

[32] De Mattos-Arruda L., Mayor R., Ng C. K. Y. (2015). Cerebrospinal fluid-derived circulating tumour DNA better represents the genomic alterations of brain tumours than plasma. *Nature Communications*.

[33] Wang Y., Springer S., Mulvey C. L. (2015). Detection of somatic mutations and HPV in the saliva and plasma of patients with head and neck squamous cell carcinomas. *Science Translational Medicine*.

[34] Melkonyan H. S., Feaver W. J., Meyer E. (2008). Transrenal nucleic acids: from proof of principle to clinical tests. *Annals of the New York Academy of Sciences*.

[35] Mandel P., Metais P. (1948). Les acides nucleiques du plasma sanguin chez l'homme. *Comptes Rendus des Seances de la Societe de Biologie et de Ses Filiales*.

[36] Thierry A. R., El M. S., Gahan P. B., Anker P., Stroun M. (2016). Origins, structures, and functions of circulating DNA in oncology. *Cancer Metastasis Reviews*.

[37] Leon S. A., Shapiro B., Sklaroff D. M., Yaros M. J. (1977). Free DNA in the serum of cancer patients and the effect of therapy. *Cancer Research*.

[38] Breitbach S., Sterzing B., Magallanes C., Tug S., Simon P. (2014). Direct measurement of cell-free DNA from serially collected capillary plasma during incremental exercise. *Journal of Applied Physiology*.

[39] De Vlaminck I., Martin L., Kertesz M. (2015). Noninvasive monitoring of infection and rejection after lung transplantation. *Proceedings of the National Academy of Sciences of the United States of America*.

[40] Tsai N. W., Lin T. K., Chen S. D. (2011). The value of serial plasma nuclear and mitochondrial DNA levels in patients with acute ischemic stroke. *Clinica Chimica Acta*.

[41] Rodrigues F. E., Simon D., Ikuta N. (2014). Elevated cell-free plasma DNA level as an independent predictor of mortality in patients with severe traumatic brain injury. *Journal of Neurotrauma*.

[42] De Vlaminck I., Valantine H. A., Snyder T. M. (2014). Circulating cell-free DNA enables noninvasive diagnosis of heart transplant rejection. *Science Translational Medicine*.

[43] Stroun M., Anker P., Maurice P., Lyautey J., Lederrey C., Beljanski M. (1989). Neoplastic characteristics of the DNA found in the plasma of cancer patients. *Oncology*.

[44] Bardelli A., Pantel K. (2017). Liquid biopsies, what we do not know (yet). *Cancer Cell*.

[45] Thierry A. R., Mouliere F., El Messaoudi S. (2014). Clinical validation of the detection of *KRAS* and *BRAF* mutations from circulating tumor DNA. *Nature Medicine*.

[46] Vogelstein B., Kinzler K. W. (1999). Digital PCR. *Proceedings of the National Academy of Sciences of the United States of America*.

[47] Birkenkamp-Demtroder K., Nordentoft I., Christensen E. (2016). Genomic alterations in liquid biopsies from patients with bladder cancer. *European Urology*.

[48] Sacher A. G., Paweletz C., Dahlberg S. E. (2016). Prospective validation of rapid plasma genotyping for the detection of EGFR and KRAS mutations in advanced lung cancer. *JAMA Oncology*.

[49] Schiavon G., Hrebien S., Garcia-Murillas I. (2015). Analysis of *ESR1* mutation in circulating tumor DNA demonstrates evolution during therapy for metastatic breast cancer. *Science Translational Medicine*.

[50] Taly V., Pekin D., Benhaim L. (2013). Multiplex picodroplet digital PCR to detect KRAS mutations in circulating DNA from the plasma of colorectal cancer patients. *Clinical Chemistry*.

[51] Pekin D., Skhiri Y., Baret J. C. (2011). Quantitative and sensitive detection of rare mutations using droplet-based microfluidics. *Lab on a Chip*.

[52] Cai X., Janku F., Zhan Q., Fan J. B. (2015). Accessing genetic information with liquid biopsies. *Trends in Genetics*.

[53] Newman A. M., Lovejoy A. F., Klass D. M. (2016). Integrated digital error suppression for improved detection of circulating tumor DNA. *Nature Biotechnology*.

[54] Newman A. M., Bratman S. V., To J. (2014). An ultrasensitive method for quantitating circulating tumor DNA with broad patient coverage. *Nature Medicine*.

[55] Paweletz C. P., Sacher A. G., Raymond C. K. (2016). Bias-corrected targeted next-generation sequencing for rapid, multiplexed detection of actionable alterations in cell-free DNA from advanced lung cancer patients. *Clinical Cancer Research*.

[56] Dietz S., Schirmer U., Mercé C. (2016). Low input whole-exome sequencing to determine the representation of the tumor exome in circulating DNA of non-small cell lung cancer patients. *PLoS One*.

[57] Ulz P., Belic J., Graf R. (2016). Whole-genome plasma sequencing reveals focal amplifications as a driving force in metastatic prostate cancer. *Nature Communications*.

[58] Ulz P., Thallinger G. G., Auer M. (2016). Inferring expressed genes by whole-genome sequencing of plasma DNA. *Nature Genetics*.

[59] Frenel J. S., Carreira S., Goodall J. (2015). Serial next-generation sequencing of circulating cell-free DNA evaluating tumor clone response to molecularly targeted drug administration. *Clinical Cancer Research*.

[60] Murtaza M., Dawson S. J., Tsui D. W. Y. (2013). Non-invasive analysis of acquired resistance to cancer therapy by sequencing of plasma DNA. *Nature*.

[61] Chan K. C., Jiang P., Zheng Y. W. (2013). Cancer genome scanning in plasma: detection of tumor-associated copy number aberrations, single-nucleotide variants, and tumoral heterogeneity by massively parallel sequencing. *Clinical Chemistry*.

[62] Heitzer E., Ulz P., Belic J. (2013). Tumor-associated copy number changes in the circulation of patients with prostate cancer identified through whole-genome sequencing. *Genome Medicine*.

[63] Leary R. J., Sausen M., Kinde I. (2012). Detection of chromosomal alterations in the circulation of cancer patients with whole-genome sequencing. *Science Translational Medicine*.

[64] Khakoo S., Georgiou A., Gerlinger M., Cunningham D., Starling N. (2018). Circulating tumour DNA, a promising biomarker for the management of colorectal cancer. *Critical Reviews in Oncology/Hematology*.

[65] Tsai W., Nimgaonkar A., Segurado O. (2018). Prospective clinical study of circulating tumor cells for colorectal cancer screening. *Journal of Clinical Oncology*.

[66] Cima I., Kong S. L., Sengupta D. (2016). Tumor-derived circulating endothelial cell clusters in colorectal cancer. *Science Translational Medicine*.

[67] Sun J., Fei F., Zhang M. (2019). The role of ^m^SEPT9 in screening, diagnosis, and recurrence monitoring of colorectal cancer. *BMC Cancer*.

[68] Warren J. D., Xiong W., Bunker A. M. (2011). Septin 9 methylated DNA is a sensitive and specific blood test for colorectal cancer. *BMC Medicine*.

[69] Widschwendter M., Evans I., Jones A. (2017). Methylation patterns in serum DNA for early identification of disseminated breast cancer. *Genome Medicine*.

[70] Widschwendter M., Zikan M., Wahl B. (2017). The potential of circulating tumor DNA methylation analysis for the early detection and management of ovarian cancer. *Genome Medicine*.

[71] Guo S., Diep D., Plongthongkum N., Fung H. L., Zhang K., Zhang K. (2017). Identification of methylation haplotype blocks aids in deconvolution of heterogeneous tissue samples and tumor tissue-of-origin mapping from plasma DNA. *Nature Genetics*.

[72] Paduch R. (2016). The role of lymphangiogenesis and angiogenesis in tumor metastasis. *Cellular Oncology (Dordrecht)*.

[73] Romiti A., Raffa S., Di Rocco R. (2014). Circulating tumor cells count predicts survival in colorectal cancer patients. *Journal of Gastrointestinal and Liver Diseases*.

[74] Szczerba B. M., Castro-Giner F., Vetter M. (2019). Neutrophils escort circulating tumour cells to enable cell cycle progression. *Nature*.

[75] Mohme M., Riethdorf S., Pantel K. (2017). Circulating and disseminated tumour cells -- mechanisms of immune surveillance and escape. *Nature Reviews Clinical Oncology*.

[76] Rahbari N. N., Bork U., Schölch S. (2016). Metastatic spread emerging from liver metastases of colorectal cancer: does the seed leave the soil again?. *Annals of Surgery*.

[77] Chen K. Z., Lou F., Yang F. (2016). Circulating tumor DNA detection in early-stage non-small cell lung cancer patients by targeted sequencing. *Scientific Reports*.

[78] Yang Y. C., Wang D., Jin L. (2018). Circulating tumor DNA detectable in early- and late-stage colorectal cancer patients. *Bioscience Reports*.

[79] Misale S., Yaeger R., Hobor S. (2012). Emergence of *KRAS* mutations and acquired resistance to anti-EGFR therapy in colorectal cancer. *Nature*.

[80] Yang M., Forbes M. E., Bitting R. L. (2018). Incorporating blood-based liquid biopsy information into cancer staging: time for a TNMB system?. *Annals of Oncology*.

[81] Lack J., Gillard M., Cam M., Paner G. P., VanderWeele D. J. (2017). Circulating tumor cells capture disease evolution in advanced prostate cancer. *Journal of Translational Medicine*.

[82] Bidard F. C., Michiels S., Riethdorf S. (2018). Circulating tumor cells in breast cancer patients treated by neoadjuvant chemotherapy: a meta-analysis. *Journal of the National Cancer Institute*.

[83] Wang L., Zhou S., Zhang W. (2019). Circulating tumor cells as an independent prognostic factor in advanced colorectal cancer: a retrospective study in 121 patients. *International Journal of Colorectal Disease*.

[84] Rahbari N. N., Aigner M., Thorlund K. (2010). Meta-analysis shows that detection of circulating tumor cells indicates poor prognosis in patients with colorectal cancer. *Gastroenterology*.

[85] van Dalum G., Stam G. J., Scholten L. F. A. (2015). Importance of circulating tumor cells in newly diagnosed colorectal cancer. *International Journal of Oncology*.

[86] Tan Y., Wu H. (2018). The significant prognostic value of circulating tumor cells in colorectal cancer: a systematic review and meta-analysis. *Current Problems in Cancer*.

[87] Bork U., Rahbari N. N., Schölch S. (2015). Circulating tumour cells and outcome in non-metastatic colorectal cancer: a prospective study. *British Journal of Cancer*.

[88] Sotelo M. J., Sastre J., Maestro M. L. (2015). Role of circulating tumor cells as prognostic marker in resected stage III colorectal cancer. *Annals of Oncology*.

[89] Denève E., Riethdorf S., Ramos J. (2013). Capture of viable circulating tumor cells in the liver of colorectal cancer patients. *Clinical Chemistry*.

[90] Basnet S., Zhang Z. Y., Liao W. Q., Li S. H., Li P. S., Ge H. Y. (2016). The prognostic value of circulating cell-free DNA in colorectal cancer: a meta-analysis. *Journal of Cancer*.

[91] Parkinson C. A., Gale D., Piskorz A. M. (2016). Exploratory analysis of TP53 mutations in circulating tumour DNA as biomarkers of treatment response for patients with relapsed high-grade serous ovarian carcinoma: a retrospective study. *PLoS Medicine*.

[92] Reinert T., Scholer L. V., Thomsen R. (2016). Analysis of circulating tumour DNA to monitor disease burden following colorectal cancer surgery. *Gut*.

[93] El M. S., Mouliere F., Du Manoir S. (2016). Circulating DNA as a strong multimarker prognostic tool for metastatic colorectal cancer patient management care. *Clinical Cancer Research*.

[94] Tie J., Cohen J. D., Wang Y. (2019). Circulating tumor DNA analyses as markers of recurrence risk and benefit of adjuvant therapy for stage III colon cancer. *JAMA Oncology*.

[95] Spindler K. G., Boysen A. K., Pallisgård N. (2017). Cell-free DNA in metastatic colorectal cancer: a systematic review and meta-analysis. *The Oncologist*.

[96] Tabernero J., Lenz H. J., Siena S. (2015). Analysis of circulating DNA and protein biomarkers to predict the clinical activity of regorafenib and assess prognosis in patients with metastatic colorectal cancer: a retrospective, exploratory analysis of the CORRECT trial. *The Lancet Oncology*.

[97] Bettegowda C., Sausen M., Leary R. J. (2014). Detection of circulating tumor DNA in early- and late-stage human malignancies. *Science Translational Medicine*.

[98] Zhou J., Chang L., Guan Y. (2016). Application of circulating tumor DNA as a non-invasive tool for monitoring the progression of colorectal cancer. *PLoS One*.

[99] Douillard J. Y., Oliner K. S., Siena S. (2013). Panitumumab-FOLFOX4 treatment and RAS mutations in colorectal cancer. *The New England Journal of Medicine*.

[100] Di Nicolantonio F., Martini M., Molinari F. (2008). Wild-type *BRAF* is required for response to panitumumab or cetuximab in metastatic colorectal cancer. *Journal of Clinical Oncology*.

[101] Knebel F. H., Bettoni F., Da F. L., Camargo A. A., Sabbaga J., Jardim D. L. (2019). Circulating tumor DNA detection in the management of anti-EGFR therapy for advanced colorectal cancer. *Frontiers in Oncology*.

[102] Siravegna G., Mussolin B., Buscarino M. (2015). Clonal evolution and resistance to EGFR blockade in the blood of colorectal cancer patients. *Nature Medicine*.

[103] Heitzer E., Auer M., Gasch C. (2013). Complex tumor genomes inferred from single circulating tumor cells by array-CGH and next-generation sequencing. *Cancer Research*.

[104] Suhaimi N.-A. M., Foong Y. M., Lee D. Y. S. (2015). Non-invasive sensitive detection of *KRAS* and *BRAF* mutation in circulating tumor cells of colorectal cancer patients. *Molecular Oncology*.

[105] Kalikaki A., Politaki H., Souglakos J. (2014). KRAS genotypic changes of circulating tumor cells during treatment of patients with metastatic colorectal cancer. *PLoS One*.

[106] Carter L., Rothwell D. G., Mesquita B. (2017). Molecular analysis of circulating tumor cells identifies distinct copy-number profiles in patients with chemosensitive and chemorefractory small-cell lung cancer. *Nature Medicine*.

[107] Scholer L. V., Reinert T., Ørntoft M. B. W. (2017). Clinical implications of monitoring circulating tumor DNA in patients with colorectal cancer. *Clinical Cancer Research*.

[108] Diehl F., Schmidt K., Choti M. A. (2008). Circulating mutant DNA to assess tumor dynamics. *Nature Medicine*.

[109] Tie J., Wang Y., Tomasetti C. (2016). Circulating tumor DNA analysis detects minimal residual disease and predicts recurrence in patients with stage II colon cancer. *Science Translational Medicine*.

[110] Tie J., Cohen J. D., Wang Y. (2019). Serial circulating tumour DNA analysis during multimodality treatment of locally advanced rectal cancer: a prospective biomarker study. *Gut*.

[111] Wang Y., Li L., Cohen J. D. (2019). Prognostic potential of circulating tumor DNA measurement in postoperative surveillance of nonmetastatic colorectal cancer. *JAMA Oncology*.

[112] Reinert T., Henriksen T. V., Christensen E. (2019). Analysis of plasma cell-free DNA by ultradeep sequencing in patients with stages I to III colorectal cancer. *JAMA Oncology*.

[113] Murray D. H., Symonds E. L., Young G. P. (2018). Relationship between post-surgery detection of methylated circulating tumor DNA with risk of residual disease and recurrence-free survival. *Journal of Cancer Research and Clinical Oncology*.

[114] Wang W., Wan L., Wu S. (2018). Mesenchymal marker and LGR5 expression levels in circulating tumor cells correlate with colorectal cancer prognosis. *Cellular Oncology*.

[115] Tsai W. S., Chen J. S., Shao H. J. (2016). Circulating tumor cell count correlates with colorectal neoplasm progression and is a prognostic marker for distant metastasis in non-metastatic patients. *Scientific Reports*.

[116] Seeberg L. T., Waage A., Brunborg C. (2015). Circulating tumor cells in patients with colorectal liver metastasis predict impaired survival. *Annals of Surgery*.

[117] Arrazubi V., Mata E., Antelo M. L. (2019). Circulating tumor cells in patients undergoing resection of colorectal cancer liver metastases. Clinical utility for long-term outcome: a prospective trial. *Annals of Surgical Oncology*.

[118] Ma B., King A. D., Leung L. (2017). Identifying an early indicator of drug efficacy in patients with metastatic colorectal cancer-a prospective evaluation of circulating tumor cells, 18F-fluorodeoxyglucose positron-emission tomography and the RECIST criteria. *Annals of Oncology*.

[119] Rapado-Gonzalez O., Alvarez-Castro A., Lopez-Lopez R., Iglesias-Canle J., Suarez-Cunqueiro M. M., Muinelo-Romay L. (2019). Circulating microRNAs as promising biomarkers in colorectal cancer. *Cancers*.

[120] Lafitte M., Lecointre C., Roche S. (2019). Roles of exosomes in metastatic colorectal cancer. *American Journal of Physiology Cell Physiology*.

[121] Wang Y., Liu J., Ma J. (2019). Exosomal circRNAs: biogenesis, effect and application in human diseases. *Molecular Cancer*.

[122] Toiyama Y., Okugawa Y., Fleshman J., Richard B. C., Goel A. (2018). MicroRNAs as potential liquid biopsy biomarkers in colorectal cancer: a systematic review. *Biochimica Et Biophysica Acta Reviews on Cancer*.

[123] Balacescu O., Sur D., Cainap C. (2018). The impact of miRNA in colorectal cancer progression and its liver metastases. *International Journal of Molecular Sciences*.

[124] Best M. G., Sol N., In T. V. S. (2017). Swarm intelligence-enhanced detection of non-small-cell lung cancer using tumor-educated platelets. *Cancer Cell*.

[125] Best M. G., Sol N., Kooi I. (2015). RNA-Seq of tumor-educated platelets enables blood-based pan-cancer, multiclass, and molecular pathway cancer diagnostics. *Cancer Cell*.

[126] Thery C., Zitvogel L., Amigorena S. (2002). Exosomes: composition, biogenesis and function. *Nature Reviews Immunology*.

[127] Fu Y., Zhang L., Zhang F. (2017). Exosome-mediated miR-146a transfer suppresses type I interferon response and facilitates EV71 infection. *PLoS Pathogens*.

[128] Kral J., Korenkova V., Novosadova V. (2018). Expression profile of miR-17/92 cluster is predictive of treatment response in rectal cancer. *Carcinogenesis*.

[129] Vychytilova-Faltejskova P., Radova L., Sachlova M. (2016). Serum-based microRNA signatures in early diagnosis and prognosis prediction of colon cancer. *Carcinogenesis*.

[130] Boriachek K., Umer M., Islam M. N. (2018). An amplification-free electrochemical detection of exosomal miRNA-21 in serum samples. *The Analyst*.

[131] Zeng Z., Li Y., Pan Y. (2018). Cancer-derived exosomal miR-25-3p promotes pre-metastatic niche formation by inducing vascular permeability and angiogenesis. *Nature Communications*.

[132] Zhang Z., Xing T., Chen Y., Xiao J. (2018). Exosome-mediated miR-200b promotes colorectal cancer proliferation upon TGF-*β*1 exposure. *Biomedicine & Pharmacotherapy*.

[133] Hong B. S., Cho J. H., Kim H. (2009). Colorectal cancer cell-derived microvesicles are enriched in cell cycle-related mRNAs that promote proliferation of endothelial cells. *BMC Genomics*.

[134] Zou S. L., Chen Y. L., Ge Z. Z., Qu Y. Y., Cao Y., Kang Z. X. (2019). Downregulation of serum exosomal miR-150-5p is associated with poor prognosis in patients with colorectal cancer. *Cancer Biomarkers*.

[135] Yan S., Jiang Y., Liang C. (2018). Exosomal miR-6803-5p as potential diagnostic and prognostic marker in colorectal cancer. *Journal of Cellular Biochemistry*.

[136] Matsumura T., Sugimachi K., Iinuma H. (2015). Exosomal microRNA in serum is a novel biomarker of recurrence in human colorectal cancer. *British Journal of Cancer*.

[137] Jin G., Liu Y., Zhang J. (2019). A panel of serum exosomal microRNAs as predictive markers for chemoresistance in advanced colorectal cancer. *Cancer Chemotherapy and Pharmacology*.

[138] McDonald J. S., Milosevic D., Reddi H. V., Grebe S. K., Algeciras-Schimnich A. (2011). Analysis of circulating microRNA: preanalytical and analytical challenges. *Clinical Chemistry*.

[139] Cheng H. H., Yi H. S., Kim Y. (2013). Plasma processing conditions substantially influence circulating microRNA biomarker levels. *PLoS One*.

[140] Lai X., Wang M., McElyea S. D., Sherman S., House M., Korc M. (2017). A microRNA signature in circulating exosomes is superior to exosomal glypican-1 levels for diagnosing pancreatic cancer. *Cancer Letters*.

[141] Jin X., Chen Y., Chen H. (2017). Evaluation of tumor-derived exosomal miRNA as potential diagnostic biomarkers for early-stage non–small cell lung cancer using next-generation sequencing. *Clinical Cancer Research*.

[142] Cha D. J., Franklin J. L., Dou Y. (2015). *KRAS*-dependent sorting of miRNA to exosomes. *eLife*.

[143] Singh R., Pochampally R., Watabe K., Lu Z., Mo Y. Y. (2014). Exosome-mediated transfer of miR-10b promotes cell invasion in breast cancer. *Molecular Cancer*.

